# Aerobic Conditions and Endogenous Reactive Oxygen Species Reduce the Production of Infectious MS2 Phage by *Escherichia coli*

**DOI:** 10.3390/v13071376

**Published:** 2021-07-15

**Authors:** Guillaume Bastin, Aurélie Galmiche, François Talfournier, Hortense Mazon, Julie Challant, Maëlle Robin, Didier Majou, Nicolas Boudaud, Christophe Gantzer

**Affiliations:** 1 LCPME, CNRS, Université de Lorraine, F-54000 Nancy, France; guillaume.bastin@univ-lorraine.fr (G.B.); aurelie.galmiche@univ-lorraine.fr (A.G.); julie.challant@univ-lorraine.fr (J.C.); 2 ACTALIA, Food Safety Department, 310 Rue Popielujko, 50000 Saint Lô, France; M.Robin@actalia.eu (M.R.); n.boudaud@actalia.eu (N.B.); 3 IMoPA, CNRS, Université de Lorraine, F-54000 Nancy, France; francois.talfournier@univ-lorraine.fr (F.T.); hortense.mazon@univ-lorraine.fr (H.M.); 4 ACTIA (Association de Coordination Technique pour L’industrie Agro-Alimentaire), 16 Rue Claude Bernard, CEDEX 05, 75231 Paris, France; D.MAJOU@actia-asso.eu

**Keywords:** MS2 phage, *Escherichia coli*, infectivity, reactive oxygen species, aerobic/anaerobic conditions

## Abstract

Most of the defective/non-infectious enteric phages and viruses that end up in wastewater originate in human feces. Some of the causes of this high level of inactivity at the host stage are unknown. There is a significant gap between how enteric phages are environmentally transmitted and how we might design molecular tools that would only detect infectious ones. Thus, there is a need to explain the low proportion of infectious viral particles once replicated. By analyzing lysis plaque content, we were able to confirm that, under aerobic conditions, *Escherichia coli* produce low numbers of infectious MS2 phages (I) than the total number of phages indicated by the genome copies (G) with an I/G ratio of around 2%. Anaerobic conditions of replication and ROS inhibition increase the I/G ratio to 8 and 25%, respectively. These data cannot only be explained by variations in the total numbers of MS2 phages produced or in the metabolism of *E. coli*. We therefore suggest that oxidative damage impacts the molecular replication and assembly of MS2 phages.

## 1. Introduction

A number of different kinds of viruses are able to replicate in the human gut. Pathogenic enteric viruses (e.g., norovirus, hepatitis A virus) replicate in human cells whereas some bacteriophages (e.g., coliphages) replicate in human microbiota, especially in enterobacterial cytosol [1,2]. Both kinds of viruses are transmitted to humans via the fecal-oral route and therefore need to be able to survive in the environment, including in water and food matrices [3]. F-specific RNA bacteriophages (e.g., MS2 phage) belonging to the *Leviviridae* family share a similar structure to that of pathogenic enteric viruses. They are composed of a ~30 nm capsid protein and a single-stranded RNA genome [4,5]. These coliphages are therefore commonly used to model the behavior of pathogenic enteric viruses, evaluate the efficacy of disinfection treatments or indicate viral pollution in water and vulnerable foodstuffs [3,6,7].

After they have been excreted from the human body, these enteric viral particles (viruses and phages) are exposed to aerobic stresses in the environment. Oxidative stress is one of the main factors known to decrease their infectivity. In particular, UV and higher temperatures increase oxidative stress, thus inactivating enteric viral particles, including MS2 phages, in the environment [8,9,10,11,12]. The intentional addition of exogenous oxidants (e.g., hypochlorous acid, ozone) for disinfection purposes is also known to be an effective means of inactivating enteric viral particles, including MS2 phages [2,10,13,14,15,16]. Oxidant-dependent viral inactivation may also take place in the human gut. Although human gut lumen is primarily anaerobic and bacterial/cellular cytoplasm is a reduced environment [17,18], endogenous reactive oxygen species (ROS) are produced. Higher quantities of ROS, including enteric viruses and bacteriophages, are produced during viral infections [19,20,21,22,23]. Within host cells, viral replication—including RNA replication; the production of capsid proteins; the assembly of nucleocapsids; and the detachment of phages from the host cell—may therefore all occur in the presence of endogenous ROS.

While the impact of exogenous oxidants on the infectivity of enteric viral particles, including MS2 phages, has been extensively studied [8,11,12,13,14,15,16,24,25,26,27,28,29], little is known about the potential impact of endogenous ROS produced by host cells on the replication and infectivity of enteric viral particles. It is reasonable to suggest that endogenous oxidants can inactivate a viral particle using the same mechanism(s) as exogenous oxidants. For instance, it is well known that exogenous oxidants can alter the viral capsid or genome, thus preventing the first step in the virus’s replication cycle (recognition of the host cell receptor; release of the viral genome into the host cell; replication of the viral genome) [10,11,12,16,26,27,30,31,32,33]. Interestingly, even when it has not been able to successfully complete at least one of the early steps of its replication cycle, a MS2 phage particle that has been inactivated by oxidation has not necessarily been completely disrupted, as it remains visible under electron microscopy [2,33]. For this reason, after the application of oxidative stress, the ratio of infectious particles (I, defined by plaque-forming units) divided by genome copies (G, defined by RT-PCR) (I/G ratio) decreases by several orders of magnitude [2,11,14,27,28,34,35,36]. Notably, at the initial point of pollution (i.e., feces, raw wastewater, sludge) the I/G ratios of enteric viruses are usually very low. I/G ratios of RNA viruses of 2, 0.1 and 0.002% in fecal and water samples have been reported [37,38,39]. This suggests that most enteric viral particles released by host cells and expelled by the human gut are not infectious. We therefore need to consider both viral replication in the host cell (potentially influenced by endogenous oxidation) and the survival of viral particles outside the host cell (influenced by exogenous environmental oxidants) in order to: (i) better understand the entire transmission cycle of enteric viral particles, and (ii) develop a reliable method that can detect which viral particles are infectious [38].

This study was therefore designed to determine the potential influence of oxidative replication conditions on the production and infectivity of MS2 phages, a model that is often used to test the impact of exogenous oxidants on the infectivity of viral RNA particles. The oxidative conditions were tested by culturing MS2 phage and its host *E. coli* under both aerobic and anaerobic conditions in vitro. In the aerobic condition, which is more likely to produce endogenous ROS, the pharmacological tools VAS-2870 and EUK-134 were used to limit the presence of endogenous ROS and produce a significant increase in the infectivity of newly replicated MS2 phages. The extent of MS2 phage replication and host cell growth were determined by measuring plaques and colonies, respectively. We then monitored single plaque-forming units (PFU) by quantifying infectious phages by culture and genome copies using RT-qPCR to observe any changes in I/G ratios under these experimental conditions.

## 2. Materials and Methods

### 2.1. Production and Purification of Phage Suspension

MS2 phage suspension was prepared using a standard procedure previously described [40]. Briefly summarized: we replicated *E. coli* K-12 Hfr (hereafter called *E. coli*) host cells in liquid broth. The non-lysed bacteria were excluded by filtration through a 0.22 µm-pored membrane. The phages were then concentrated by ultracentrifugation and purified by cesium chloride gradient density centrifugation. Finally, cesium chloride was removed through two successive dialyses in 1 mM PBS (13.7 mM NaCl, 0.27 mM KCl, 1 mM Na_2_HPO_4_, 0.18 mM KH_2_PO_4_ at pH 7.4) using Float-A-Lyzer G2 with Biotech Cellulose Ester membranes (MWCO: 100 kDa, volume: 1 mL, Spectra/Por®, Spectrum Laboratories, CA, USA). The solution was stirred gently at 4 °C. The dialysis suspension was quantified using the standard double-layered agar method (ISO-10705) [41] with *E. coli* host cells and TYGA media for the under layer (1% (*w*/*v*) tryptone, 0.1% yeast extract, 0.8% NaCl, 1.6% agar) and soft agar TYGA mixed with phage and bacterial suspension for the upper layer (1% tryptone, 0.1% yeast extract, 0.8% NaCl, 0.8% agar). The phage concentration was expressed in PFU.mL^−1^. The initial phage stock suspension was titrated at 1 × 10^14^ PFU.mL^−1^ and stored at 4 °C, in darkness.

### 2.2. Phage Replication Conditions during Plaque Formation

To obtain and analyze single lysis plaques, we expected to obtain ~100 lysis plaques formed by MS2 phages cultured on a lawn of *E. coli* for each 10 cm-diameter culture plate. An amount of 1 mL of 100 PFU.mL^−1^ suspension (10^12^- semifold dilution of 1 × 10^14^ PFU.mL^−1^ MS2 stock in 10 mM PBS at pH 7.4) was added to 2.5 mL -agar medium (boiled until fused and then kept and used at 55 °C) and 1 mL *E. coli* (precultured in aerobic conditions until it reached the early exponential phase (OD_600_ of 0.2–0.3). The resulting mixture was vortexed/homogenized and poured onto the first agar layer. The entire protocol was followed using the conditions and media described in the ISO standard procedure (ISO-10705) [41]. The plates were placed in different environmental conditions for each type of experiment. To produce anaerobic conditions, the plates were placed in an airtight chamber in which lit candles (which burned out after 1–2 min.) were used to consume the oxygen. To produce aerobic conditions, the plates were placed in a non-airtight chamber and we did not control atmospheric oxygen content in the air of the room. In all cases, the plates were incubated for 18 h at 37 °C prior to lysis plaque size measurement, phage extraction, titration and RNA quantification. Where applicable, VAS-2870 (# BML-EI395 from Enzo Life Sciences, Farmingdale, New York, USA) and EUK-134 (#10006329 from Cayman Chemical, Ann Arbor, Michigan, USA) were added to the mixture containing the phages, *E. coli* and semi-agar medium to produce final concentrations of 50 and 250 µM, respectively. VAS-2870 is a well-known pan-inhibitor of NADPH oxidases (NOXs). In the case of eukaryotic NOXs, the inhibition is due to the covalent modification of a conserved catalytic domain cysteine [42,43]. EUK-134 is a membrane-permeable manganese salen complex that mimics superoxide dismutase and catalase and can therefore scavenge ROS from within an *E. coli* compound [44,45]. DMSO and H_2_O were used to solubilize VAS-2870 and EUK-134 respectively and therefore served as controls.

### 2.3. Extraction of MS2 Phages from Lysis Plaques

The diameters of individual lysis plaques were first measured using a mm ruler and the plaques were then carefully excised using a scalpel and placed in 2 mL of 10 mM PBS, pH 7.4 in a sterile 6 mL glass tube. They were then vortexed at maximum stirring speed to resuspend MS2 phages released from lysis plaques. The optimal vortexing duration was determined to be 3 intervals of 4 s for 2 mm plaques and 3 intervals of 16 s for 3–5 mm plaques. This resuspension of lysis plaques in 2 mL of PBS was used as the “benchmark” suspension to compare infectious phages defined using the double-layered agar method with genomes defined by RT-qPCR. This resuspension step was arbitrarily considered to constitute a dilution of 1/10.

### 2.4. Quantification of Infectious Phages in Individual Lysis Plaques

To quantify the number of infectious phages in each lysis plaque, we followed ISO procedure [41]. Briefly summarized: suspensions of phages extracted from lysis plaques (as described above) were quantified using the lysis plaque titration technique. This involved decreasing the concentrations of the phage suspensions to an estimated ~1000, 100, 10, 1 and 0 PFU.mL^−1^ through successive dilutions in 10 mM PBS at pH 7.4. An amount of 1 mL of each diluted suspension was combined with 2.5 mL of semi-agar medium and 1 mL of *E. coli* preculture and poured onto a first layer of agar medium. The culture was then incubated for 18 h at 37 °C under aerobic conditions prior to quantification. For these 1 mL inoculation volumes, the titration of each sample expressed in PFU.mL^−1^ corresponds to the number of (lysis plaques/per plate)*(1/dilution (10^−x^)).

A total of 6 plaques were quantified, covering all culture conditions, 2 lysis plaques per size and per experiment. The mean and standard errors of the 6 samples were calculated.

### 2.5. Quantification of MS2-RNA in Plaques, Using RT-qPCR

The concentration of MS2-RNA in each titrated sample of infectious MS2 phages was estimated as ~10 times higher than that of the infectious titer. On the basis of these estimates, each sample was diluted to a concentration that produced ~1 × 10^5^ RNA copies of MS2.mL^−1^ using 10 mM PBS at pH 7.4 in order to stay within a reasonable concentration range (with exponentials at 20–25 qPCR cycles) in order to perform RT-qPCR under optimum conditions, as follows. Five hundred µL of the suspensions were brought in contact with 1000 µL of lysis buffer (bioMérieux, Marcy l’Etoile, France, 280134) to extract the RNA from the capsids. The RNA was then purified using NucliSens EasyMag (bioMérieux, Marcy l’Etoile, France), following the manufacturer’s instructions. The RNA was eluted in 100 µL of elution buffer and stored at −80 °C. In the experiment using VAS-2870 and EUK-134, heat extraction was also used to extract the RNA (to confirm the yields obtained by the extraction method described above). This involved placing the 500 µL samples in an Eppendorf Thermomixer (Eppendorf, Hamburg, Germany) at 95 °C for 2 min at 300 rpm and then immediately placing the samples on ice (for ~10 min) and then plating them for RT-qPCR.

Reverse transcription and qPCR were performed using the RNA UltraSense™ One-Step Quantitative RT-PCR System (#11732927, Thermo Fisher Scientific, Waltham, Massachusetts, USA) following the manufacturer’s recommendations with a few modifications, using 20 pmol of each primer and 6 pmol of the probe in a 20 µL reaction volume. The reaction was carried out using a StepOne Plus real-time PCR system (Thermo Fisher Scientific, Waltham, Massachusetts) for 30 min at 50 °C (reverse transcription) and 5 min at 95 °C (hot start), followed by 45 cycles of 15 s at 95 °C (denaturation) and 40 s at 58 °C (annealing/extension). We used the following primers and probe, developed by Ogorzaly et al. [46]: TCGATGGTCCATACCTTAGATGC (positions 1255–1277 on sequence MS2; Genbank accession number NC 001417), and ACCCCGTTAGCGAAGTTGCT (positions 1404–1423). The probe sequence was FAM-CTCGTCGACAATGG-MGBNFQ (positions 1362–1375). Quantification was carried out in duplicate using a standard curve range of 5 × 10^−1^–5 × 10^−4^ gc/reaction, which was determined using ddPCR (as described below). Once RT-qPCR is complete, the standard curve provides an equation of the type y = ax + b, which allows the number of RNA copies per sample to be determined using the following equation: 10^((Ct sample − b)/(a)).^

### 2.6. MS2-RNA Quantification Using Digital Droplet PCR to Establish the Standard Curve for RT-qPCR

A digital droplet PCR (ddPCR) was performed to quantify the MS2 genome and establish standards for RT-qPCR quantification. We used the primers and probe described above [46].

Amplifications were performed in a 20 μL reaction mixture containing 5 μL of the template RNA, and 15 μL of the One-Step RT-ddPCR™ kit for probes (Bio-Rad, Hercules, CA, USA). The reaction mix contained 0.9 μM of each primer and 0.3 μM of the probe. The samples were placed in the droplet generator using 70 μL of generator oil. Picoliter droplet emulsions (40 μL) were then transferred to a Veriti 96-well thermal cycler (Thermo Fisher Scientific, Waltham, MA, USA). MS2 RNA amplification was performed under the following conditions: reverse transcription was performed using a 60 min hold at 50 °C, followed by a 10 min hold at 95 °C, 40 cycles of 30 s at 95 °C, 60 s at 60 °C and finally, a 10 min hold at 98 °C. The ramp rate between each step was set at 70%. After amplification, the plate was transferred to a QX100TM droplet reader (Bio-Rad, Hercules, CA, USA) using QuantaSoft™ software (Bio-Rad, Hercules, CA, USA) to measure the number of positive and negative droplets on the basis of their fluorescence amplitude. The genome concentrations obtained using this method are expressed in genome copies (gc) per unit volume.

### 2.7. Visualization of E. coli Growth on Semi-Agar Media

This experiment was designed to allow us to infer estimated host cell growth from host cell density on the bacterial lawn, under different growth conditions. The growth of the *E. coli* colonies was considered to indicate how the *E. coli* grew on the bacterial lawn. To achieve this, we set up the culture plates in a manner similar to ISO-10705 [41], except that no phages were inoculated and *E. coli* was spread on top of, instead of inside, the semi-agar media. An amount of 4.5 mL of the semi-agar medium was poured on top of the first agar layer. The semi-agar medium was left to solidify at room temperature for 10 min, after which the culture plates were placed in a room air incubator for 1 h at 37 °C to desiccate slightly, prior to the addition of *E. coli*. We aimed to obtain ~125 CFU (colony-forming units) of *E. coli* per culture plate. The *E. coli* was therefore precultured in Erlenmeyer flasks for 2 h and rotated at 150 rpm in a 37 °C water bath. The *E. coli* concentration was experimentally predetermined as ~5 × 10^7^ CFU·mL^−1^ under these conditions (importantly, this pre-culture is similar to the ones used to inoculate the bacterial lawn with MS2 phages to form *E. coli* lysis plaques). This *E. coli* concentration was diluted 2 × 10^5^-fold using 10 mM PBS at pH 7.4 at 20 °C. An amount of 250 µL of the diluted culture was then spread onto the layer of semi-agar media. The culture was incubated for 18 h at 37 °C in a room air incubator or an airtight chamber in which the oxygen was consumed by lit candles (which burned out after 1–2 min) for aerobic or anaerobic cultures, respectively. Where applicable, VAS-2870 (# BML-EI395 from Enzo Life Sciences, Farmingdale, New York, USA) and EUK-134 (#10006329 from Cayman Chemical, Ann Arbor, Michigan, USA) were added to semi-agar media to achieve final concentrations of 250 µM. DMSO and H_2_O were used to solubilize VAS-2870 and EUK-134 respectively, and were therefore used as controls. The controls were subjected to similar treatments, carried out on the same days as the sample experiments. Pictures were captured using a 13-megapixel (Samsung) camera with automatic adjustment. Measurements of the circumferences of the *E. coli* colonies were generated using ImageJ 1.48v.

### 2.8. Data Analysis

All statistical analyses were performed using R statistical software (Rx64 v.3.5.3). The Shapiro-Wilk test was performed with an alpha level of 0.05 to check the normality of the data. If the data set followed a normal distribution (*p* > 0.05), parametric tests were applied. A paired or unpaired sample Student’s *t*-test was performed on the dependent or independent data, respectively, that followed a normal distribution. Non-parametric tests were used for data sets with a non-normal distribution (*p* < 0.05). A Wilcoxon signed-rank test or Mann–Whitney *U* test was applied to dependent or independent data with a non-normal distribution. The significance level was set to 0.05 for all tests.

## 3. Results

### 3.1. Aerobic Culture Conditions Decrease the Production of Infectious MS2 Phages

**Figure 1:**
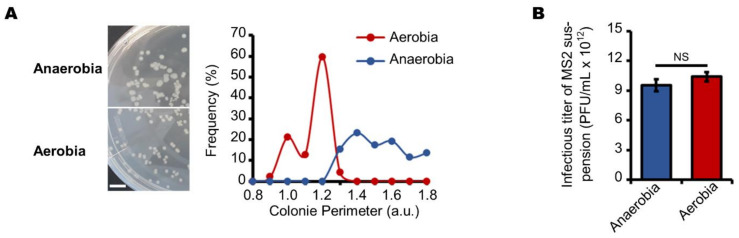
Aerobic and anaerobic culture conditions affect *E. coli* growth without changing its susceptibility to MS2 phage infection. (**A**) *E. coli* colonies grown under anaerobic conditions are larger than those grown under aerobic conditions. The left panels show pictures of *E. coli* colonies grown on double layer agarose plates under anaerobic and aerobic conditions for 18 h at 37 °C (scale bar corresponds to 1.0 cm). The data displayed in the right panel show the circumference distribution of 50 *E. coli* colonies, cultured under anaerobic (blue) and aerobic conditions (red) (“a.u.” stands for arbitrary unit). (**B**) the same MS2 suspension induced the formation of similar numbers of plaques when cultured on a lawn of *E. coli* under anaerobic and aerobic conditions. In all panels, the data are representative of experiments carried out on separate days ((**A**): *n* = 2; (**B**): *n* = 3). Error bars indicate standard errors. “NS” stands for statistically non-significant: *p* > 0.05, the statistical tests are described in the Materials and Methods section.

Aerobic and anaerobic growth conditions have been shown to alter energy metabolism and morphology of *E. coli* [47,48,49,50,51]. We therefore wondered to what extent these growth conditions would alter *E. coli* growth within our experimental setup. We observed that *E. coli* colonies grown on agar plates are larger under anaerobic than under aerobic conditions (Figure 1A). The circumference index of the colonies increased from 1.0–1.3 under aerobic to 1.3–1.8 (a.u.) under anaerobic conditions. These data indicate that *E. coli* may saturate more rapidly the agarose network when grown in a lawn under anaerobic conditions. Based on these preliminary observations, we evaluated the way in which changing host cell growth conditions affect host cells’ ability to replicate MS2 phages. We therefore first tested whether the PFU.mL^−1^ of MS2 in the suspension titer differed using the NF EN ISO 10705-1 2001 method [41] which is performed under aerobic conditions compared to under anaerobic conditions. However, we found that the MS2 phage suspension titer was unchanged at 1 × 10^13^ PFU.mL^−1^ under these conditions (Figure 1B). These data show that when cultured on a lawn, *E. coli* could replicate infectious MS2 particles under both aerobic and anaerobic conditions. We then investigated the content of the plaques resulting from PFU replication under aerobic and anaerobic conditions.

**Figure 2:**
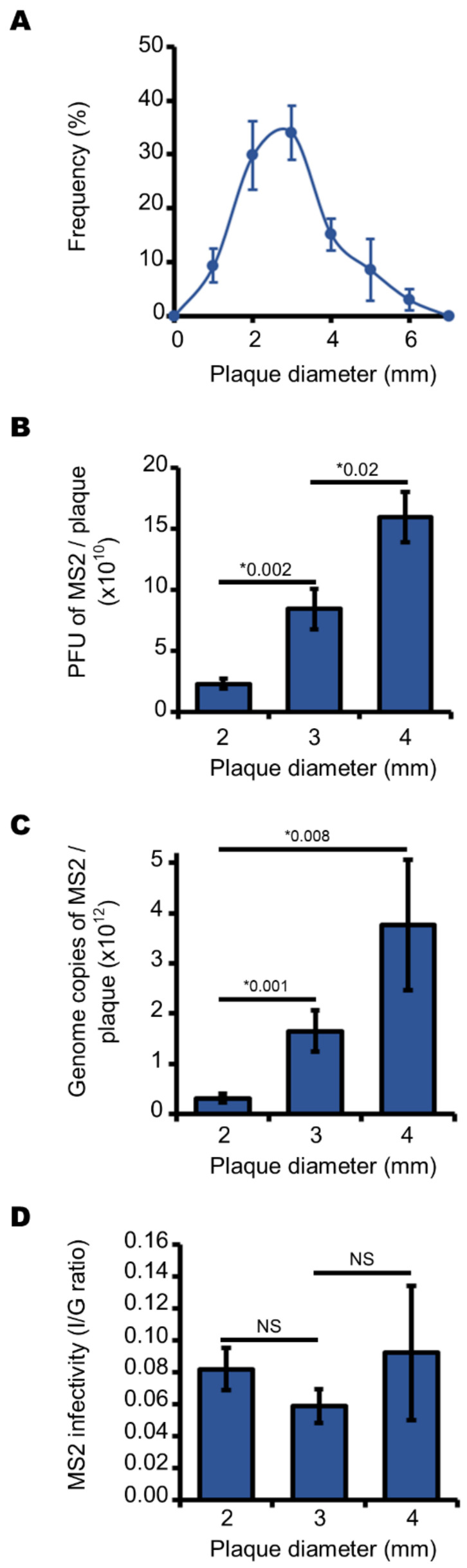
Larger plaques contain more infectious MS2 phages and genomes, but all plaques contain MS2 phages with similar levels of infectivity when cultured under anaerobic conditions. (**A**) curve showing the normal distribution of the plaques’ diameter (in mm) formed by infectious MS2 phages cultured on a lawn of *E. coli*. (**B**) histogram showing the increasing number of infectious MS2 phages depending on plaque diameter, ranging from 2.0 to 4.0 mm. (**C**) histogram showing the increasing number of MS2 genome copies depending on plaque diameter, ranging from 2.0 to 4.0 mm. (**D**) histogram showing the stable I/G ratio of infectious particles (I) divided by the number of MS2 genome copies (G) depending on plaque diameter, ranging from 2.0 to 4.0 mm. Each data point is an average of several replicates (*n* = 7 for panel (**A**); *n* = 6 for panels (**B**–**D**)) obtained on three separate days. Error bars indicate standard errors. “*” stands for “*p* value” determined as described in the Materials and Methods section. “NS” stands for statistically non-significant: *p* > 0.05.

We first investigated the MS2 phage plaques formed by *E. coli* at 37 °C under anaerobic conditions, as these conditions more closely approximate those of the human gut, where the bacteria normally replicate in vivo. We observed that the diameters of the plaques obtained under these conditions followed a normal distribution: the most frequent diameters were 2, 3 and 4 mm in size (Figure 2A). We wondered what determined this variability in size. To find out, we analyzed the viral contents of 2, 3 and 4 mm plaques and found that mean values of 2.3 × 10^10^, 8.9 × 10^10^, and 1.5 × 10^11^ infectious MS2 phages were quantified for plaques of 2, 3 and 4 mm in diameter, respectively (Figure 2B). These data show that the larger the plaques, the more infectious MS2 phages they contained (I) (Figure 2B). Similarly, the larger the plaques the more phage genome copies they contained (G) (Figure 2C). Together, these data suggest that variation in plaque size is not correlated with the infectivity of the phages (I/G) in the plaques. Indeed, the I/G ratio was independent of the diameter of the plaques: it was statistically similar in plaques of 2–4 mm diameter (Figure 2D). Together, these data show that infected *E. coli* yielded ~92% of the non-infectious MS2 phages, in anaerobic cultures (Figure 2D).

**Figure 3:**
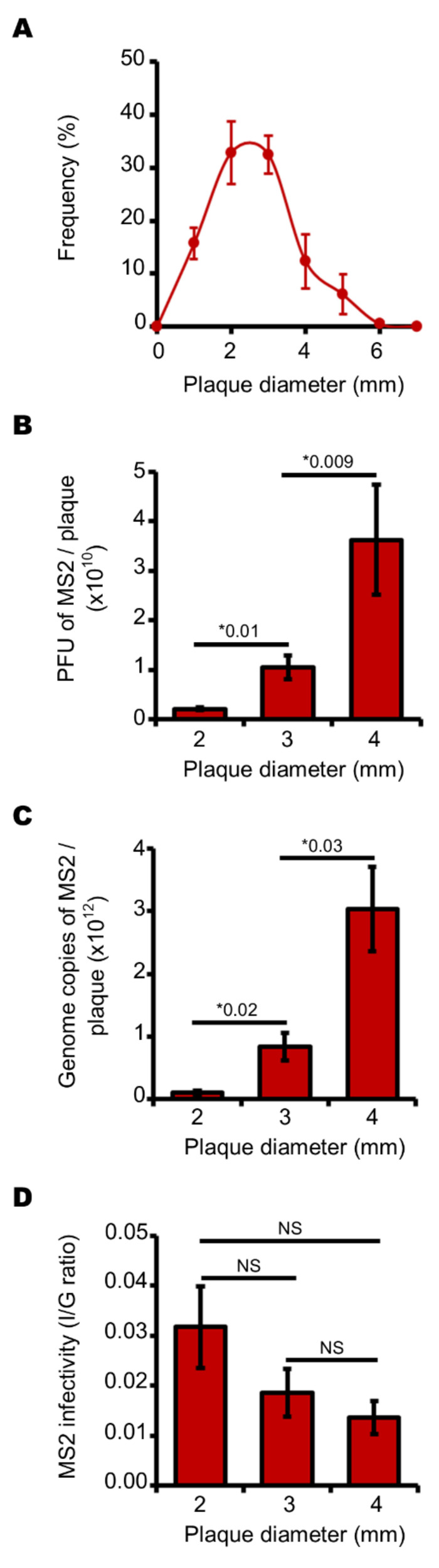
Larger plaques contain more infectious MS2 phages and genomes but phages are slightly less infectious in larger plaques when cultured under aerobic conditions. (**A**) curve showing the normal distribution of plaque diameter (in mm) formed by infectious MS2 phages cultured on a lawn of *E. coli*. (**B**) histogram showing the increasing number of infectious MS2 phages depending on plaque diameter, ranging from 2.0 to 4.0 mm. (**C**) histogram showing the increasing number of MS2 genome copies depending on plaque diameter, ranging from 2.0 to 4.0 mm. (**D**) histogram showing the slightly decreasing I/G ratio of infectious particles (I) divided by the number of MS2 genome copies (G) depending on plaque diameter, ranging from 2.0 to 4.0 mm. Each data point is an average of several replicates (*n* = 7 for panel **A**; *n* = 6 for panels **B** to **D**) obtained on three separate days. Error bars indicate standard errors. “*” stands for “p value” determined as described in the Materials and Methods section. “NS” stands for statistically non-significant: *p* > 0.05.

We then studied MS2 phage plaques formed by *E. coli* under aerobic conditions at 37 °C by simply changing the incubation conditions of the culture plates. The diameters of the plaques also followed a normal distribution and fell within a similar range of sizes (1–5 mm) to those produced under anaerobic conditions (Figure 3A). As we had observed under anaerobic conditions, larger plaques contained more infectious phages. Mean values of 2.1 × 10^9^, 1.2 × 10^10^ and 3.7 × 10^10^ infectious MS2 phages were quantified for plaques of 2, 3 and 4 mm in diameter, respectively (Figure 3B). These data confirmed that plaque size is correlated with quantity of infectious phages, under these culture conditions. Similarly, larger plaques contained more MS2 phage genome copies (Figure 3C). Here too, an increase in plaque diameter was not correlated with an increase in the I/G ratio which was not statistically different in plaques ranging from 2 to 4 mm in diameter (Figure 3D). Together, these data show that infected *E. coli* yielded ~98% of non-infectious MS2 phages in aerobic cultures (Figure 3D).

**Figure 4:**
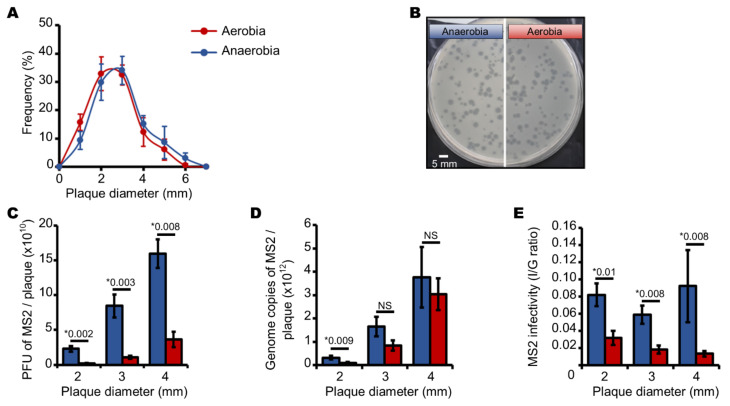
Aerobic and anaerobic culture conditions affect MS2 phage plaque size and content. (**A**) curve showing the normal distribution of plaque diameter (in mm) formed by infectious MS2 phages cultured on a lawn of *E. coli*. (**B**) pictures of plaques of MS2 phages cultured on a lawn of *E. coli* (white scale bar corresponds to 5 mm). (**C**) histogram showing the increasing number of infectious MS2 phages depending on plaque diameter, ranging from 2.0 to 4.0 mm. (**D**) histogram showing the increasing number of MS2 genome copies depending on plaque diameter, ranging from 2.0 to 4.0 mm. (**E**) histogram showing the slightly decreasing I/G ratio of infectious particles (I) divided by the number of MS2 genome copies (G) depending on plaque diameter, ranging from 2.0 to 4.0 mm. Each data point is an average of several replicates (*n* = 7 for panel (**A**); *n* = 6 for panels (**B**–**D**)) obtained on three separate days. “*” stands for “p value” determined as described in the Materials and Methods section. “NS” stands for statistically non-significant: *p* > 0.05.

We should recall that *E. coli* grew in larger colonies on solid media under anaerobic compared to under aerobic conditions (Figure 1A). At first, it seemed that this did not impact the replication of MS2 phages because the mean sizes and distributions of plaques cultivated under aerobic and anaerobic conditions were similar (Figure 4A,B). However, the contents of the plaques differed. The number of infectious phages decreased significantly when MS2 phages were produced under aerobic conditions (Figure 4C). Plaques of 2–4 mm in diameter grown under aerobic conditions contained about ten-fold fewer infectious phages than similar-sized plaques grown under anaerobic conditions (Figure 4C). There were also only half as many MS2 phage genome copies in 2–3 mm plaques grown under aerobic conditions as there were in similar-sized plaques grown under anaerobic conditions (Figure 4D), although the number of MS2 phage genome copies in 4 mm plaques were statistically similar under anaerobic and aerobic conditions (Figure 4D). The I/G ratios were therefore significantly lower when MS2 phages were replicated in *E. coli* under aerobic conditions (Figure 4E). The proportion of infectious MS2 phages decreased from ~8% under anaerobic conditions to ~2% under aerobic conditions, regardless of plaque size (Figure 4E).

Together, these data show that oxidative culture conditions decreased bacterial growth and negatively impacted the production of infectious MS2 phages released by *E. coli* cultured on a lawn.

### 3.2. Endogenous Reactive Oxygen Species Produced by E. coli Decrease the Production of Infectious MS2 Phages

**Figure 5:**
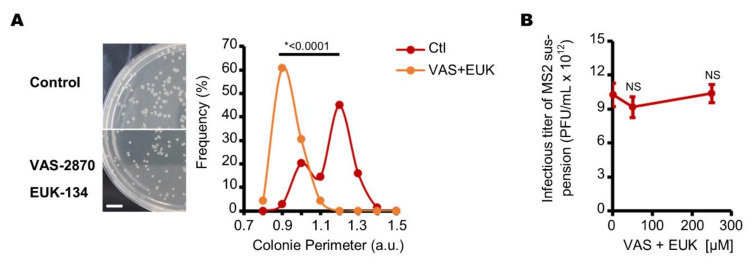
*E. coli* colonies are smaller when cultured with VAS-2870 and EUK-134 under aerobic conditions, without changing its susceptibility to infection by MS2 phages. (**A**) *E. coli* colonies are smaller when grown with VAS-2870 and EUK-134 compared to control, under aerobic conditions. The left panels show pictures of *E. coli* colonies grown on double layer agarose plates for 18 h at 37 °C (scale bar corresponds to 1.0 cm). The data displayed in the right panel show the circumference distribution of 70 *E. coli* colonies cultured with VAS-2870 and EUK-134 (orange) and control (red) (“a.u.” stands for arbitrary unit). (**B**) the same MS2 suspension induced the formation of similar-sized plaques when cultured on a lawn of *E. coli* under aerobic conditions with or without VAS-2870 (50 or 250 µM) and EUK-134 (50 or 250 µM). In all panels, the data are representative of experiments carried out on separate days ((**A**): *n* = 2; (**B**): *n* = 4). Error bars indicate standard errors. “NS” stands for statistically non-significant: *p* > 0.05, the statistical tests are described in the Materials and Methods section.

The NADPH oxidase (NOX) family of proteins are known to be major producers of ROS [42,43,52]. Interestingly, a gene that codes a NOX was recently identified in the DNA of *E. coli* [53]. Although it is not yet known whether the corresponding protein is constitutively expressed, the structure of this NOX catalytic domain is predicted to be similar to those in human cells [53]. We therefore hypothesized that the NOX family pan inhibitor (VAS-2870) [42,43] would also reduce the prevalence of endogenous ROS in this assay. Because *E. coli* can also produce endogenous ROS through intracellular molecular mechanisms such as fumarate reductase and aspartate oxidase [54,55,56], we decided to combine VAS-2870 with a ROS scavenger (EUK-134) that functions as a superoxide dismutase and catalase [44] to test the potential impact of endogenous ROS on MS2 phage replication. First, we observed that the *E. coli* colonies grown on agar plates under aerobic conditions were smaller when VAS-2870 and EUK-134 were used (Figure 5A). In particular, the colonies’ circumference indices decreased from 1.0–1.3 to 0.8–1.1 (a.u.) with the use of VAS-2870 and EUK-134. This suggests that VAS-2870 and EUK-134 slow *E. coli* growth or change its morphology under aerobic conditions. On the strength of these observations, we tested whether these compounds changed the host cells’ capacity to replicate MS2 phages. However, under the same conditions, no significant difference in the MS2 phage suspension titer was observed (Figure 5B). These data suggest that when cultured on a lawn under aerobic conditions, *E. coli* can replicate similarly infectious MS2 particles with or without VAS-2870 and EUK-134. To challenge this result further, we then investigated the content of the plaques resulting from PFU replication under aerobic conditions both with and without VAS-2870 and EUK-134.

**Figure 6:**
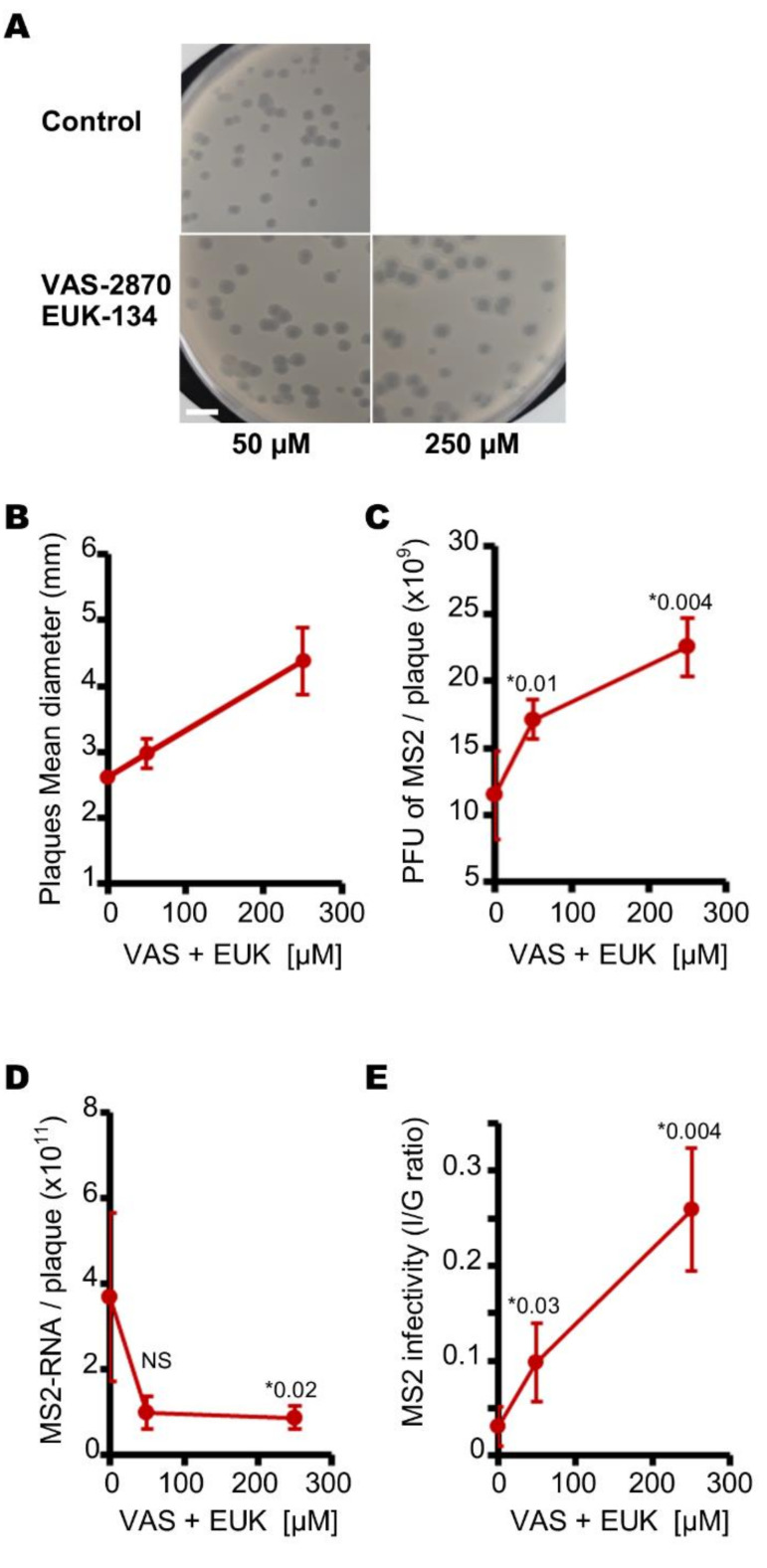
VAS-2870 and EUK-134 increase the size of MS2 plaques and the infectivity of MS2 phages when cultured under aerobic conditions with *E. coli*. (**A**) pictures of plaques of MS2 phages cultured on a lawn of *E. coli* under aerobic conditions with or without VAS-2870 and EUK-134, at concentrations of 50 or 250 µM (white scale bar corresponds to 1.0 cm). (**B**) the presence of VAS-2870 and EUK-134 in concentrations of 0 and 250 µM increases the size of MS2 plaques when cultured on a lawn of *E. coli* under aerobic conditions. (**C**) curve showing the evolution of the amount of infectious MS2 phages in 3 mm plaques depending on the concentration of VAS-2870 and EUK-134. (**D**) curve showing the change in number of MS2 genome copies in 3 mm plaques depending on the concentration of VAS-2870 and EUK-134. E, VAS-2870 and EUK-134 at concentrations of 0 and 250 µM increase the infectivity of MS2 phages (I/G ratio) in 3 mm plaques cultured under aerobic conditions. Each data point is an average of several replicates (*n* = 7 for panels A to E) obtained on four separate days. Error bars indicate standard errors. “*” stands for “p value” determined as described in the Materials and Methods section. “NS” stands for statistically non-significant: *p* > 0.05.

We previously showed that plaque size was correlated with levels of infectious phage (Figure 4C). We therefore evaluated the difference in plaque size induced by the presence of VAS-2870 and EUK-134. The presence of VAS-2870 and EUK-134, at concentrations of 50 and 250 µM, respectively, increases the mean diameters of plaques from 2.6 mm to 3.0 and 4.4, respectively (Figure 6A,B). Analyses of the 3-mm plaques revealed consistent ~1.5- and ~2-fold increases in the number of infectious phages in the presence of 50 and 250 µM of ROS inhibitors, respectively (Figure 6C). Although these plaques contained more infectious MS2 phages, they contained around four times fewer genome copies than similarly sized plaques formed in the absence of VAS-2870 and EUK-134 (Figure 6D). The MS2 phage I/G ratios replicated by *E. coli* on the lawn increased from 3 to ~10 and ~25% in the presence of 50 and 250 µM of both VAS-2870 and EUK-134, respectively (Figure 6E). Together, these data suggest that endogenous ROS decrease phage infectivity in plaques in aerobic cultures.

Taken together, these results provide a fuller picture of how oxidative culture conditions and endogenous ROS impact the production of MS2 phages yielded by *E. coli* cultured on a lawn. In particular, they clearly show that these conditions affect the number of genome copies in plaques and, most importantly, the quantity of infectious particles. It appears that the less oxidative the cultures’ conditions, the more infectious the MS2 particles are. Aerobic conditions induced the production of fewer infectious MS2 phages than anaerobic conditions. By contrast, under aerobic conditions, the compounds VAS-2870 and EUK-134 can significantly increase the infectivity of MS2 phages. Together, these data may partly explain the observed low infectivity of enteric viral particles in the environment and therefore they need to be tested with more experimental models.

## 4. Discussion 

This study aimed to discover whether oxidative conditions and endogenous ROS can influence the infectivity of MS2 phages produced by *E. coli* host cells. A production yield included the total number of genome copies and of infectious particles. We opted to use PFU-forming plaques in solid media colonized by a lawn of host cells because this approach allows for tracing the replication of individual infectious particles. This type of culture has been described as a complex, natural simulated biofilm environment [57].

With only a few exceptions, plaques attain a maximum width/diameter after a period of incubation specific to the phage, host cell and conditions [57]. The MS2 phage can only infect *E. coli* during the early exponential growth phase, during which the bacteria produce F-pili, due to the high levels of available ATP [58]. In the stationary phase, decreasing ATP availability stops *E. coli* from producing F-pili, thus blocking the entry of MS2 phages and therefore preventing infection and replication. As a consequence, plaque size remains stable during *E. coli*’s stationary growth phase, which corresponds to the incubation time used in our study (i.e., 18 h). Interestingly, plaque size can also be affected by a number of parameters affecting viral and host cells [57,59] such as the virus’s diffusivity in the medium (without a host cell); viral adsorption onto the host cell; the virus’s latent replication period; burst size (the number of viral particles expelled per host cell/viral replication cycle); and host cell density on the lawn. In addition, viral genome mutations can modify capsid protein sequences, which can alter diffusivity in the medium and thus plaque size [59]. All our experiments were carried out on a single stock of viruses, so that we could rule out major variations in capsid diffusivity. The agar and nutrient content of the media also remained the same in all tested conditions; therefore, this parameter cannot explain the phenotypes observed. We will therefore now present an integrated overview of all remaining parameters by defining the ratio of the production yield of infectious phages (I) to the total number of phage genomes (G): the I/G ratio.

Aerobic and anerobic environments were compared. Oxygen was shown to alter *E. coli* metabolism [47,56,60,61] and also *E. coli* cell length [51] in a way that could decrease colony size. Our results consistently show that *E. coli* grow in larger colonies on solid media under anaerobic conditions. We therefore hypothesized that plaque size should decrease as a result of the increase in the density of the host cells on the lawn, if no other parameter was modified [57]. However, the size and distribution of plaques remained the same under aerobic and anaerobic conditions. These data suggest that the increase in the numbers of infectious phages under anaerobic conditions probably offset increased *E. coli* growth, resulting in plaques of similar size to those formed under aerobic conditions. Given that genome replication remains constant overall, we speculate that the MS2 phages may be inactivated by modifications, perhaps molecular [33], that arise during viral particle morphogenesis or after host cell lysis. In any case, our data show that aerobic conditions yield lower percentages of infectious viral particles than anaerobic conditions. This suggests that, to study infectious phages, phage replication and cell tropism, we should produce F-specific RNA phages in vitro under anaerobic conditions if the host cells are anaerobic facultative. This may apply to more models. These results should inspire for change of laboratory customs knowing that aerobic-flasks culture for phages and aerobic chambers culture for eukaryotic cells and virus culture are more convenient, making them generally used instead of anaerobic conditions closer to the in vivo reality.

Since higher levels of endogenous ROS were reported during infection by enteric viral particles [19,20,21,22,23], it was logical to test whether they affected MS2 phage infectivity. We chose to test endogenous ROS under aerobic conditions, mainly because MS2 phages were less infectious under aerobic conditions than under anaerobic conditions. Moreover, even though endogenous ROS can be produced in *E. coli* under anaerobic conditions [54], aerobic conditions are intrinsically more oxidative. In this assay, ROS production was inhibited by using VAS-2870 to simultaneously inhibit NADPH oxidases [52] and using EUK-134 to mimic greater expression of SOD and catalases [44,45]. Our results show that these two ROS inhibitors decreased *E. coli* growth on solid/agar-containing media. Others have previously shown that a decrease in the density of host cells on the lawn increases plaque size if all other parameters remain unchanged [57]. Therefore, it was unsurprising that plaque size increased in the presence of VAS-2870 and EUK-134. VAS-2870 and EUK-134 affected viral replication by slightly decreasing the quantity of MS2 genome. Although the influence of ROS on MS2 phage replication in *E. coli* remains unclear, some authors have demonstrated that ROS is directly involved in the RNA replication of the tobacco mosaic virus in eukaryotic plant cells, thus facilitating the spread of the virus [62,63]. In naked RNA viruses, such as hepatitis C, greater intracellular oxidative stress seems to facilitate host cell lysis, viral exit and therefore the spread of pathology [19,64,65]. In contrast, MS2 phages probably do not require ROS to exit *E. coli* since phage RNA codes for a functional lysis protein [66]. VAS-2870 and EUK-134 consistently failed to decrease the production of infectious MS2 phages under our experimental conditions. These data show that endogenous ROS is not necessarily crucial to the spread of the virus. Moreover, VAS-2870 and EUK-134 highly increased MS2 infectivity (I/G) in plaques in this experimental setup. Together, the decrease in MS2 genome copies (G) and increase in infectious MS2 phages (I) suggest that endogenous ROS affects the final phases of replication (i.e., capsid assembly). While exogenous ROS have already demonstrated the capacity to inactivate already formed enteric viral particles, it is still unclear at which stages endogenous ROS inactivate or prevent the activation of enteric viral particles, given that they can be present within host cells at every step of the viral replication.

Taken as a whole, our study demonstrates that the presence of oxidants (ROS or aerobic conditions) decreases production of MS2 phages by *E. coli* by decreasing both the total number of particles produced (RNA production) and—even more significantly—by decreasing the production of infectious MS2 phage. Further studies are required to help achieve a better understanding the role of endogenous ROS in the replication and spread of viral particles. The importance of oxidative conditions during replication can be also further investigated. If the right virus/phage and host cell models are used, it may lead to a better understanding of the relationship between host cell and viral spreading. In the context of understanding the whole replication cycle of enteric viruses and phages, this work could highlight the prevalence of inactivated viral particles in the environment and therefore show that, in order to assess infection risk, we should develop ways of measuring only the *infectious* viral particles in food and water exposed to possible fecal pollution.

## Data Availability

Data are available upon request.
